# High pulse wave velocity is associated with enlarged perivascular spaces in dementia with Lewy bodies

**DOI:** 10.1038/s41598-024-64984-8

**Published:** 2024-06-17

**Authors:** Naoki Saji, Yoshino Kinjo, Kenta Murotani, Shumpei Niida, Akinori Takeda, Takashi Sakurai

**Affiliations:** 1 Center for Comprehensive Care and Research On Memory Disorders, Hospital, National Center for Geriatrics and Gerontology, 7-430 Morioka, Obu, Aichi 474-8511 Japan; 2 Department of Cardiovascular Medicine, Nephrology and Neurology, University of the Ryukyus Graduate School of Medicine, Nishihara-cho, Okinawa Japan; 3 Biostatistics Center, Graduate School of Medicine, Kurume University, Kurume, Fukuoka Japan; 4 Research Institute, National Center for Geriatrics and Gerontology, Obu, Aichi Japan; 5 Department of Prevention and Care Science, Research Institute, National Center for Geriatrics and Gerontology, Obu, Aichi Japan; 6 Department of Cognition and Behavioral Science, Nagoya University Graduate School of Medicine, Nagoya, Aichi Japan

**Keywords:** Cerebral small vessel disease, Cognitive decline, DLB, Enlarged perivascular spaces, Pulse wave velocity, Dementia, Neurology, Risk factors, Neuroscience, Cognitive neuroscience

## Abstract

Previous studies have demonstrated associations between enlarged perivascular spaces (EPVS) and dementias such as Alzheimer’s disease. However, an association between EPVS and dementia with Lewy bodies (DLB) has not yet been clarified. We performed a cross-sectional analysis of our prospective study cohort of 109 participants (16 with DLB). We assessed cognitive function, pulse wave velocity (PWV), and brain magnetic resonance imaging features. The relationships between EPVS and DLB were evaluated using multivariable logistic regression analyses. Compared with the non-dementia group, the DLB group was more likely to have EPVS in the basal ganglia. Compared with participants without EPVS, those with EPVS were older and had cognitive impairment and high PWV. In multivariable analyses, EPVS in the basal ganglia was independently associated with DLB. High PWV was also independently associated with EPVS in both the basal ganglia and centrum semiovale. High PWV may cause cerebrovascular pulsatility, leading to accelerated EPVS in DLB participants.

## Introduction

Dementia is a substantial healthcare problem worldwide^1^. Globally, 46.8 million people have dementia, and the number of individuals with dementia and its associated costs are expected to increase^1,2^. Because Japan is facing a similar healthcare problem, an integrated dementia research program has been introduced to reduce the social burden caused by dementia^3^.

Brain magnetic resonance imaging (MRI) has been widely used to improve the diagnosis of dementia-related disorders and better understand the cause of cognitive impairment in older patients^4^. In particular, cerebral small vessel disease (SVD), which is detectable on brain MRI scans, is an important risk factor of dementia^2,4^.

Enlarged perivascular spaces (EPVS) are a component of cerebral SVD. Perivascular spaces are compartments surrounding cerebral blood vessels that become visible on MRI when enlarged. EPVS is suggested to reflect dysfunctional perivascular clearance of soluble waste products from the brain^5^, which indicates glymphatic dysfunction^6^. Recent studies have suggested positive associations between (1) EPVS and Alzheimer’s disease (AD)^5^, and (2) EPVS and Parkinson’s disease^7^. However, associations between EPVS and dementia with Lewy bodies (DLB) have not yet been analyzed.

DLB is similar to Parkinson’s disease and is the second most common neurodegenerative dementia^8^. DLB is characterized by visual hallucinations, cognitive impairment, sleep disturbance, parkinsonism, and autonomic dysfunction^8^. There is currently no cure for DLB; treatments are aimed at ameliorating specific symptoms^9^. An assessment of DLB from the perspective of EPVS may contribute to a better understanding of the pathophysiology of this disease.

We are currently conducting an observational study regarding cognitive function and the gut microbiota. In previous studies, we have revealed that gut microbial dysregulation is associated with cognitive decline^10–12^ and cerebral SVD^13,14^. We are also planning to evaluate the association between gut microbiota and DLB because recent studies have assessed such a relationship^15,16^. In the present study, however, we aimed to evaluate the relationship between DLB and cerebral SVD (in particular, EPVS). We believe that an examination of cerebral SVD and the links between cerebral SVD, gut microbiota, and DLB may clarify as-yet-unidentified associations between gut microbiota and DLB.

In the present study, we thus evaluated the relationships between EPVS and DLB by performing a sub-analysis of the data from our ongoing clinical study. We hypothesized that EPVS would be associated with DLB, similar to the previously reported association between EPVS and AD.

## Results

### Participant characteristics

We analyzed 109 participants in this sub-analysis (women: 49%, mean age: 76 years, normal cognition [NC]: *n* = 17; mild cognitive impairment [MCI]: *n* = 76; DLB: *n* = 16). Women were less likely than men to have a smoking habit (women vs. men; 5.6% vs. 41.1%, *p* < 0.001) or consume alcohol (22.6% vs. 55.4%, *p* < 0.001). However, there were no significant differences between women and men in age (median age; 76 vs. 77 years, *p* = 0.944), global cognitive function (Mini-Mental State Examination [MMSE] score; 26 vs. 26, *p* = 0.734; Clinical Dementia Rating Scale-Sum of Boxes [CDR-SB]; 1.8 vs. 1, *p* = 0.072), and risk factors such as hypertension (58.5% vs. 64.3%, *p* = 0.560) and diabetes mellitus (15.1% vs. 16.1%, *p* = 1.000).

### DLB versus non-dementia

Compared with participants without DLB, those with DLB tended to have hypertension (DLB vs. non-DLB; 87.5% vs. 57.0%, *p* = 0.025), a history of fall (73.3% vs. 34.4%, *p* = 0.009), lower nutritional status (median Mini-Nutritional Assessment-Short Form score; 9 vs. 13, *p* < 0.001), and lower cognitive function (Tables 1, S1). Participants with DLB were also more likely than those without DLB to have EPVS in the basal ganglia (BG-PVS; 62.5% vs. 24.7%, *p* = 0.006), higher plasma neurofilament light chain (NfL) concentrations (median value: 31.4 vs. 21.3 pg/mL, *p* = 0.001), and higher scores in the voxel-based specific regional analysis system for Alzheimer’s Disease (VSRAD; median score: 1.05 vs. 0.84, *p* = 0.020). There were no significant differences in EPVS in the centrum semiovale (CS-PVS) between participants with and without DLB (31.3% vs. 35.5%, *p* = 1.000).

**Table 1:** Comparison of background information between participants with DLB and those without dementia.

	DLB ( +)	DLB (-)	*p*
	(*n* = 16)	(*n* = 93)	
Demographics			
Age, years	79, 73–82	76, 68–80	0.092
Sex, female, n (%)	5 (31.3)	48 (51.6)	0.178
Risk factors			
Hypertension, n (%)	14 (87.5)	53 (57.0)	0.025
Diabetes mellitus, n (%)	5 (31.3)	12 (12.9)	0.127
APOE ε4 carrier, n (%)	6 (37.5)	21 (22.6)	0.219
Comprehensive geriatric assessment			
Barthel Index	88, 68–100	100, 100–100	< .0001
IADL impairment, n (%)	13 (81.3)	28 (30.1)	< .001
DBDS	12, 9–23	6, 3–11	0.001
History of fall in 1 year	11 (73.3)	31 (34.4)	0.009
MNA-SF	9, 6–9	13, 11–13	< .0001
Cognitive function			
MMSE	22, 17–24	27, 23–29	< .0001
CDR-SB	4, 2–10	1, 0.5–2	< .0001
ADAS-cog	13, 9.8–18.1	7, 4.7–10.4	0.001
RCPM	23, 19–29	29, 26–32	0.013
FAB	10, 4.8–11.3	12, 10–14	0.006
LM-WMSR I	5, 0–8	12, 7–19	0.002
LM-WMSR II	1, 0–3	5, 1–12	0.007
Brain MRI findings			
SLI, n (%)	3 (18.8)	4 (4.3)	0.064
WMH, n (%)	4 (25.0)	23 (24.7)	1.000
CMB, n (%)	5 (31.3)	14 (15.1)	0.150
BG-PVS ≥ 2, n (%)	10 (62.5)	23 (24.7)	0.006
CS-PVS ≥ 3, n (%)	5 (31.3)	33 (35.5)	1.000
VSRAD	1.05, 0.90–1.57	0.84, 0.56–1.26	0.020
Arterial stiffness			
Ankle brachial index	1.07, 1.00–1.17	1.12, 1.07–1.15	0.168
Pulse wave velocity, m/s	19.9, 17.7–21.4	17.9, 15.7–22.0	0.189
Laboratory findings			
BNP, pg/mL	40, 31.1–85.0	29.0, 14.4–59.5	0.042
NfL, pg/mL	31.4, 21.7–59.0	21.3, 15.0–26.3	0.001

Data are presented as medians, interquartile ranges or number of patients (%). The Wilcoxon rank-sum test and χ^2^ test were used.

Note that participants without dementia presented with normal cognition or mild cognitive impairment.

ADAS-cog, Alzheimer’s Disease Assessment Scale-Cognitive Subscale; APOE, apolipoprotein E; BG-PVS, enlarged perivascular spaces in the basal ganglia; BNP, brain natriuretic peptide; CDR-SB, Clinical Dementia Rating-Sum of Boxes; CMB, cerebral microbleed; CS-PVS, enlarged perivascular spaces in the centrum semiovale; DBDS, Dementia Behavior Disturbance Scale; DLB, dementia with Lewy bodies; FAB, Frontal Assessment Battery; IADL, instrumental activities of daily living; LM-WMSR, Logical Memory subtests I and II of the Wechsler Memory Scale-Revised; MMSE, Mini-Mental State Examination; MNA-SF, Mini-Nutritional Assessment-Short Form; MRI, magnetic resonance imaging; NfL, neurofilament light chain; RCPM, Raven’s Coloured Progressive Matrices; SLI, silent lacunar infarct; VSRAD, voxel-based specific regional analysis system for Alzheimer’s disease; WMH, white matter hyperintensity.

### High versus low BG-PVS scores

Compared with participants with low BG-PVS scores (EPVS score < 2), those with high BG-PVS scores (EPVS score ≥ 2) tended to be older (high vs. low BG-PVS scores; median age; 79 vs. 75 years, *p* < 0.001) and were more likely to have a history of stroke (18.2% vs. 2.6%, *p* = 0.009), higher plasma NfL concentrations (median value: 26.1 vs. 19.8 pg/mL, *p* < 0.001), and impaired cognitive function (Tables 2, S2).

**Table 2:** Comparison of background information between participants with high and low BG-PVS scores.

	BG-PVS ≥ 2	BG-PVS < 2	*p*
	(*n* = 33)	(*n* = 76)	
Demographics			
Age, years	79, 76–82	75, 67–79	< 0.001
Sex, female, n (%)	14 (42.4%)	39 (51.3%)	0.413
Risk factors			
Hypertension, n (%)	25 (75.8)	42 (55.3)	0.055
Diabetes mellitus, n (%)	8 (24.2)	9 (11.8)	0.149
APOE ε4 carrier, n (%)	10 (30.3)	17 (22.4)	0.470
Comprehensive geriatric assessment			
Barthel Index	100, 86–100	100, 100–100	0.005
IADL impairment, n (%)	20 (60.6)	21 (27.6)	0.002
DBDS	11, 5–19	6, 3–10	0.001
Vitality index	9, 9–10	10, 9–10	0.032
Cognitive function			
MMSE	24, 22–27	27, 23–29	0.031
CDR-SB	2, 1–4	1, 0.5–2	0.001
ADAS-cog	9.3, 5–13	7, 4.7–10.4	0.130
RCPM	26, 22–30	30, 27–33	0.002
FAB	10, 9–12	13, 10–14	< 0.001
LM-WMSR I	9, 4.5–14	12, 7–20	0.072
LM-WMSR II	3, 1–7	5, 1–13	0.152
Brain MRI findings			
SLI, n (%)	5 (15.2)	2 (2.6)	0.026
WMH, n (%)	9 (27.3)	18 (23.7)	0.810
CMB, n (%)	8 (24.2)	11 (14.5)	0.273
CS-PVS ≥ 3, n (%)	19 (57.6)	19 (25.0)	0.002
VSRAD	1.01, 0.77–1.21	0.84, 0.55–1.60	0.197
Arterial stiffness			
Ankle brachial index	1.08, 1.05–1.15	1.13, 1.07–1.16	0.204
Pulse wave velocity, m/s	20.3, 18.2–23.4	17.3, 15.1–21.1	0.010
Laboratory findings			
BNP, pg/mL	34.5, 25.8–86.3	28.9, 14.0–49.9	0.013
NfL, pg/mL	26.1, 21.3–35.5	19.8, 14.4–25.0	< 0.001

Data are presented as medians, interquartile ranges or number of patients (%). The Wilcoxon rank-sum test and χ^2^ test were used.

Note that participants with high BG-PVS were defined as presenting with enlarged perivascular spaces in the basal ganglia (scores ≥ 2 based on an MRI scan at the level of the basal ganglia).

ADAS-cog, Alzheimer’s Disease Assessment Scale-Cognitive Subscale; APOE, apolipoprotein E; BG-PVS, enlarged perivascular spaces in the basal ganglia; BNP, brain natriuretic peptide; BP, blood pressure; CDR-SB, Clinical Dementia Rating-Sum of Boxes; CMB, cerebral microbleed; CS-PVS, enlarged perivascular spaces in the centrum semiovale; DBDS, Dementia Behavior Disturbance Scale; FAB, Frontal Assessment Battery; IADL, instrumental activities of daily living; LM-WMSR, Logical Memory subtests I and II of the Wechsler Memory Scale-Revised; MMSE, Mini-Mental State Examination; MRI, magnetic resonance imaging; NfL, neurofilament light chain; RCPM, Raven’s Coloured Progressive Matrices; SLI, silent lacunar infarct; VSRAD, voxel-based specific regional analysis system for Alzheimer’s disease; WMH, white matter hyperintensity.

### High versus low CS-PVS scores

Compared with participants with low CS-PVS scores (EPVS score < 3), those with high CS-PVS scores (EPVS score ≥ 3) were less likely to have a smoking habit (high vs. low CS-PVS scores, 10.5% vs. 31.0%, *p* = 0.019); they also tended to have higher PWV (median value: 20.4 vs. 17.4 m/s, *p* = 0.006) and plasma NfL concentrations (median value: 24.5 vs. 20.7 pg/mL, *p* = 0.049, Tables 3, S3).

**Table 3:** Comparison of background information between participants with high and low CS-PVS scores.

	CS-PVS ≥ 3	CS-PVS < 3	*p*
	(*n* = 38)	(*n* = 71)	
Demographics			
Age, years	77, 74–80	75, 68–81	0.146
Sex, female, n (%)	17 (44.7)	36 (50.7)	0.688
Risk factors			
Hypertension, n (%)	22 (57.9)	45 (63.4)	0.680
Diabetes mellitus, n (%)	7 (18.4)	10 (14.1)	0.587
Smoking habits, n (%)	4 (10.5)	22 (31.0)	0.019
Comprehensive geriatric assessment			
Barthel Index	100, 100–100	100, 100–100	0.290
IADL impairment, n (%)	19 (50.0)	22 (31.0)	0.063
DBDS	8, 5–14	6, 3–11	0.099
Cognitive function			
MMSE	26, 23–29	25, 23–29	0.868
CDR-SB	1.3, 0.5–2.6	1, 0.5–2.6	0.537
ADAS-cog	8.2, 4.2–11.4	7.6, 5.3–11.4	0.654
RCPM	29, 24–31.3	30, 25.5–33	0.215
FAB	11, 10–14	12, 10–14	0.178
LM-WMSR I	10, 6–18	11, 6–18	0.752
LM-WMSR II	3, 1–10.5	5, 1–11	0.551
Brain MRI findings			
SLI, n (%)	2 (5.3)	5 (7.0)	1.000
WMH, n (%)	10 (26.3)	17 (23.9)	0.818
CMB, n (%)	7 (18.4)	12 (16.9)	1.000
BG-PVS ≥ 2, n (%)	19 (50.0)	14 (19.7)	0.002
VSRAD	0.94, 0.68–1.21	0.87, 0.57–1.63	0.953
Arterial stiffness			
Ankle brachial index	1.08, 1.05–1.18	1.12, 1.07–1.15	0.594
Pulse wave velocity, m/s	20.4, 17.8–23.5	17.4, 15.6–20.5	0.006
Laboratory findings			
BNP, pg/mL	30.2, 20.6–63.8	30, 13.5–64.7	0.308
NfL, pg/mL	24.5, 20.9–31.0	20.7, 15.2–28.9	0.049

Data are presented as medians, interquartile ranges or number of patients (%). The Wilcoxon rank-sum test and χ^2^ test were used.

Note that participants with high CS-PVS were defined as presenting with enlarged perivascular spaces in the centrum semiovale (scores ≥ 3 based on an MRI scan at the level of the centrum semiovale).

ADAS-cog, Alzheimer’s Disease Assessment Scale-Cognitive Subscale; APOE, apolipoprotein E; BG-PVS, enlarged perivascular spaces in the basal ganglia; BNP, brain natriuretic peptide; CDR-SB, Clinical Dementia Rating-Sum of Boxes; CMB, cerebral microbleed; CS-PVS, enlarged perivascular spaces in the centrum semiovale; DBDS, Dementia Behavior Disturbance Scale; DLB, dementia with Lewy bodies; FAB, Frontal Assessment Battery; IADL, instrumental activities of daily living; LM-WMSR, Logical Memory subtests I and II of the Wechsler Memory Scale-Revised; MMSE, Mini-Mental State Examination; MRI, magnetic resonance imaging; NfL, neurofilament light chain; RCPM, Raven’s Coloured Progressive Matrices; SLI, silent lacunar infarct; VSRAD, voxel-based specific regional analysis system for Alzheimer’s disease; WMH, white matter hyperintensity.

### PVS, PWV, and cognitive function

**Figure 1:**
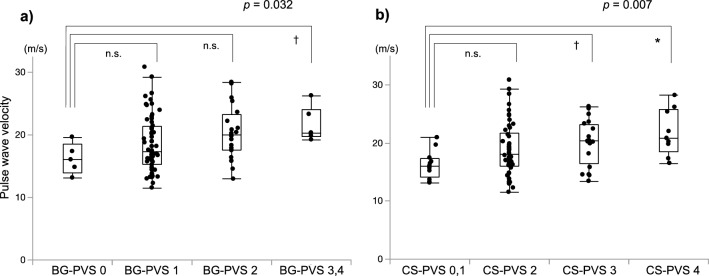
Associations between pulse wave velocity and enlarged perivascular spaces (EPVS) in the basal ganglia (BG-PVS) and centrum semiovale (CS-PVS). Here, pulse wave velocity is plotted relative to the degree of BG-PVS (**a**) and CS-PVS (**b**), stratified into four groups based on the following classifications: 0 (no EPVS), 1 (< 10 EPVS), 2 (11–20 EPVS), 3 (21–40 EPVS), and 4 (> 40 EPVS). Pulse wave velocity increased with increasing degrees of EPVS (Kruskal–Wallis test). Post hoc analysis was performed using the Steel test. *p < 0.01; ^†^*p* < 0.05; n.s., not significant.

**Figure 2:**
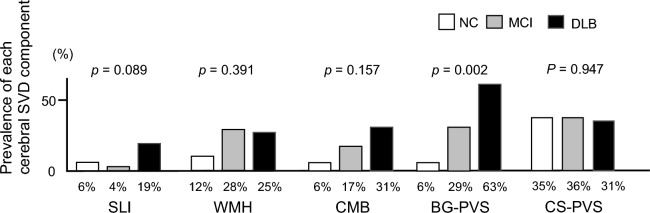
Prevalence of each component of cerebral small vessel disease stratified by cognitive function (normal cognition [NC), mild cognitive impairment [MCI), and dementia with Lewy body [DLB]). Here, the prevalence of different components of cerebral small vessel disease are plotted relative to cognitive function. The following components of cerebral small vessel disease are shown: silent lacunar infarct (SLI), white matter hyperintensity (WMH), cerebral microbleeds (CMB), and enlarged perivascular spaces in the basal ganglia (BG-PVS) and centrum semiovale (CS-PVS). Analyses were performed using the χ^2^ test.

**Figure 3:**
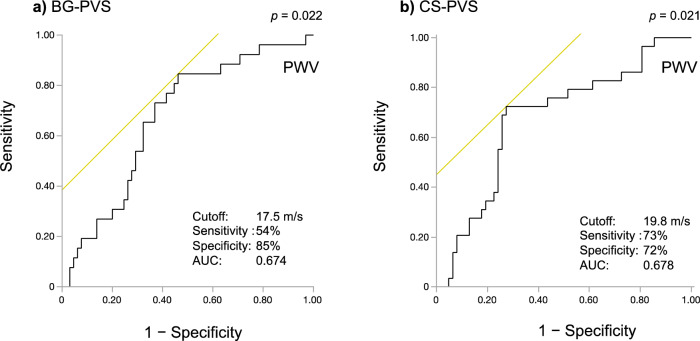
Receiver operating characteristic curves of brachial-ankle pulse wave velocity (PWV) for the detection of enlarged perivascular spaces in the basal ganglia (BG-PVS) and centrum semiovale (CS-PVS). Here, the brachial-ankle PWV cut-off values for the detection of BG-PVS (EPVS score ≥ 2) (**a**) and CS-PVS (EPVS score ≥ 3) (**b**) are shown. AUC: area under the curve.

Greater PWV was observed with greater numbers of EPVS in both of the analyzed regions (BG-PVS: *p* = 0.032, CS-PVS: *p* = 0.007; Kruskal–Wallis test, Fig. 1). The prevalence of BG-PVS (EPVS score ≥ 2) gradually increased with increasing cognitive impairment (NC vs. MCI vs. DLB: 6% vs. 29% vs. 63%, *p* = 0.002); however, similar significant associations were not detected for CS-PVS (EPVS score ≥ 3, Fig. 2). The PWV cut-off values for BG-PVS (EPVS score ≥ 2) and CS-PVS (EPVS score ≥ 3) detection were 17.5 m/s and 19.8 m/s, respectively (Fig. 3).

### Multivariable analyses

Multivariable logistic regression analyses revealed that BG-PVS was independently associated with the presence of DLB after adjusting for age, sex, PWV, risk factors, and the presence of cerebral SVD (odds ratio [OR], 95% confidence interval [CI] 4.29, 1.36–13.6, *p* = 0.012, Table 4); CS-PVS was not significantly associated with the presence of DLB. Furthermore, multivariable logistic regression analyses revealed that high PWV (PWV ≥ 19 m/s, which was the median value of the enrolled participants) was independently associated with the presence of both BG-PVS (OR 95% CI 3.84, 1.36–13.6, *p* = 0.031, Table 5) and CS-PVS (OR 95% CI 7.08, 2.32–21.6, *p* < 0.001, Table 6).

**Table 4:** Multivariable logistic regression analyses for the presence of DLB.

	OR	95% CI	*p*
BG-PVS ≥ 2*			
Model 1	5.07	1.66–15.5	0.004
Model 2	4.85	1.36–17.3	0.012
Model 3	4.80	1.26–18.3	0.018
Model 4	5.07	1.66–15.5	0.004
Model 5 (full model)			
BG-PVS ≥ 2	4.29	1.36–13.6	0.012
Dyslipidemia	3.09	0.86–11.2	0.071
Cerebral microbleeds	3.20	0.79–13.0	0.108

The dependent variable was the presence of DLB.

Note that BG-PVS ≥ 2 was defined as having a score ≥ 2 based on a magnetic resonance imaging scan at the level of the basal ganglia.

Model 1: univariate analysis. Model 2: adjusted for age, sex, and pulse wave velocity (≥ 19 m/s). Model 3: adjusted for model 2 factors and components of cerebral small vessel disease (silent lacunar infarcts, white matter hyperintensity, and cerebral microbleeds). Model 4: backward stepwise multivariable logistic regression analysis adjusted for model 2 factors, education years, and risk factors (hypertension, dyslipidemia, diabetes mellitus, ischemic heart disease, a history of stroke, chronic kidney disease, smoking, alcohol consumption, and apolipoprotein E ε4). Model 5: backward stepwise multivariable logistic regression analysis adjusted for model 3 and 4 factors.

BG-PVS, enlarged perivascular spaces in the basal ganglia; CI, confidence interval; DLB, dementia with Lewy bodies; OR, odds ratio.

**Table 5:** ORs of PWV in multivariable logistic regression analyses for the presence of BG-PVS.

	OR	95% CI	*p*
PWV ≥ 19 m/s			
Model 1	4.64	1.70–12.6	0.002
Model 2	3.11	1.06–9.07	0.034
Model 3	3.75	1.12–12.5	0.026
Model 4 (full model)			
PWV ≥ 19 m/s	3.84	1.36–13.6	0.031
Sex (female)	0.10	0.02–0.58	0.004
Age, per year	1.17	1.05–1.32	< .001
Smoking habit	0.03	0.01–0.29	< .001
Chronic kidney disease	0.04	0.01–0.32	< .001
Silent lacunar infarcts	13.8	1.66–115.1	0.010

The dependent variable was the presence of BG-PVS (score ≥ 2 based on a magnetic resonance imaging scan at the level of the basal ganglia).

Model 1: univariate analysis. Model 2: adjusted for age and sex. Model 3: backward stepwise multivariable logistic regression analysis adjusted for model 2 factors and risk factors (hypertension, dyslipidemia, diabetes mellitus, ischemic heart disease, a history of stroke, chronic kidney disease, smoking, and alcohol consumption). Model 4: backward stepwise multivariable logistic regression analysis adjusted for model 3 factors and components of cerebral small vessel disease (silent lacunar infarcts, white matter hyperintensity, and cerebral microbleeds).

BG-PVS, enlarged perivascular spaces in the basal ganglia; CI, confidence interval; OR, odds ratio; PWV, pulse wave velocity.

**Table 6:** ORs of PWV in multivariable logistic regression analyses for the presence of CS-PVS.

	OR	95% CI	*p*
PWV ≥ 19 m/s			
Model 1	4.77	1.82–12.5	< .001
Model 2	3.93	1.42–10.9	0.007
Model 3	7.08	2.32–21.6	< .001
Model 4 (full model)			
PWV ≥ 19 m/s	7.08	2.32–21.6	< .001
Smoking habit	0.23	0.06–0.90	0.022
Dyslipidemia	0.33	0.11–0.99	0.039

The dependent variable was the presence of CS-PVS (score ≥ 3 based on a magnetic resonance imaging scan at the level of the centrum semiovale).

Model 1: univariate analysis. Model 2: adjusted for age and sex. Model 3: backward stepwise multivariable logistic regression analysis adjusted for model 2 factors and risk factors (hypertension, dyslipidemia, diabetes mellitus, ischemic heart disease, a history of stroke, chronic kidney disease, smoking, and alcohol consumption). Model 4: backward stepwise multivariable logistic regression analysis adjusted for model 3 factors and components of cerebral small vessel disease (silent lacunar infarcts, white matter hyperintensity, and cerebral microbleeds).

CI, confidence interval; CS-PVS, enlarged perivascular spaces in the centrum semiovale; OR, odds ratio; PWV, pulse wave velocity.

### BG-PVS in DLB

Of the participants with DLB, participants with BG-PVS were more likely than those without BG-PVS to have greater cognitive impairment, a history of fall, higher Movement Disorder Society-Unified Parkinson’s Disease Rating Scale (UPDRS) scores, higher PWV, and higher concentrations of plasma NfL; however, age was nearly equal between the two groups (Table S4).

## Discussion

The main finding of the present study was that BG-PVS was independently associated with the presence of DLB. Furthermore, the prevalence of EPVS—both BG-PVS and CS-PVS—gradually increased with increasing cognitive impairment. We also found that higher PWV was independently associated with the presence of EPVS (both BG-PVS and CS-PVS). These findings may help to reveal the potential associations and related mechanisms between EPVS and dementia—in particular DLB—and suggest the important role of PWV in these associations.

This is the first report to demonstrate associations between EPVS and PWV (more specifically, brachial-ankle PWV) in participants with DLB. Although several studies^17,18^ have reported associations between aortic PWV and EPVS, these studies did not include participants with dementia. Thus, this novel link between EPVS and PWV in DLB participants provides insights into the natural history of dementia, and in particular DLB. We have previously reported that brachial-ankle PWV, an indicator of arterial stiffness, is associated with silent lacunar infarct (SLI)^19^, white matter hyperintensity (WMH)^20^, and progressive acute lacunar infarcts^21^; it can also predict future stroke following acute lacunar infarcts^22^. However, the ankle-brachial index was not associated with EPVS in the present study. This finding is reasonable because the ankle-brachial index indicates large-artery atherosclerosis^23^, which may be different from microvascular dysfunction. Our present data are therefore in line with the findings of previous studies and fill a knowledge gap regarding cerebral SVD—in particular EPVS—and cerebrovascular pulsatility.

Recently, EPVS have been considered a component of the glymphatic system^2,6^. The glymphatic system is an emerging hypothetical system that connects perivascular spaces, allowing the exchange of waste products between the cerebrospinal fluid surrounding the brain and the interstitial fluid within the brain parenchyma^2^. The glymphatic system plays a vital role in the clearance of several metabolic products, such as amyloid-β and tau^2^. When speculating on glymphatic dysfunction, cerebrovascular pulsatility—the driving force for cerebrospinal fluid flow within the perivascular spaces—has been advocated^24^. Our finding of an association between PWV and EPVS supports the relationship between the glymphatic system and cerebrovascular pulsatility because high PWV increases cerebral microvascular pulsation^25^.

Cerebrovascular pulsatility may also play an important role in hypertensive cerebral SVD. Pathological findings of WMH have been described as wall-thickened arteries, large perivascular spaces, and extensive arterial pulsations^26^. Cerebrovascular pulsatility (as detected by transcranial Doppler ultrasound) is reportedly associated with WMH^27^. We have previously proposed a “tsunami effect in the brain” as a potential mechanism of PWV in cerebrovascular pulsatility^25^. Thus, the presence of EPVS suggests overflowing compartments surrounding cerebral blood vessels and dysfunctional perivascular clearance, followed by a tsunami effect in the brain microvascular system.

In the present study, we found that BG-PVS were more common in participants with DLB. Furthermore, DLB participants with BG-PVS had more impaired motor and cognitive function and higher UPDRS scores. Motor dysfunction may be affected by cerebral parenchyma degeneration in the basal ganglia, as indicated by BG-PVS. A previous study reported that BG-PVS is associated with cognitive impairment and Parkinson’s disease but that CS-PVS is not associated with cognitive impairment in Parkinson’s disease participants^7^. Furthermore, higher PWV is reportedly associated with more severe dementia in DLB participants^28^. These findings are in line with the present data. Our findings are also reasonable because DLB tends to complicate autonomic dysfunction^8^, leading to blood pressure variability. Blood pressure variability is a risk of both dementia^29,30^ and cerebral SVD^31,32^. Thus, our findings suggest a multiplex linkage among PWV, EPVS, and cognitive impairment in DLB. Because we did not include participants with AD or Parkinson’s disease in the present study, the mechanism of this multiplex linkage will need to be investigated in more detail in future studies.

Another notable finding of the present study was that NfL was significantly associated with DLB, as has been previously reported^33^. However, CS-PVS was not as strongly associated with cognitive impairment in DLB as has been previously reported^7^. This discrepancy may be the result of the present study setting—in which we mainly enrolled participants with DLB as the dementia group—because CS-PVS is strongly associated with cerebral amyloid angiopathy^34^. In addition, we found that participants with BG-PVS were older and more likely to have hypertension, suggesting the presence of hypertensive cerebral SVD, as has been previously reported^34^. Lastly, the cut-off value of PWV for the detection of BG-PVS (17.5 m/s) was nearly the same as that reported for other subtypes of cerebral SVD, such as SLI (17.2 m/s)^19^, progressive acute lacunar stroke (18.2 m/s)^21^, and WMH (18.3 m/s)^20^. These findings are therefore also reasonable because these factors are all components of hypertensive cerebral SVD.

The present study has several strengths. First, we revealed novel relationships among EPVS, PWV, and DLB. High PWV was strongly associated with EPVS, suggesting that high PWV increases cerebrovascular pulsatility, thus accelerating both hypertensive cerebral SVD and glymphatic dysfunction. Second, we revealed that BG-PVS was associated with DLB. These findings are novel because, although associations between DLB and various MRI findings (such as WMH or cerebral microbleeds [CMB]) have been discussed^8,35^, an association with EPVS has not been previously reported. Glymphatic dysfunction may also exist in DLB, and might accelerate α-synuclein aggregation. We offer new research directions regarding cerebral SVD (specifically, the glymphatic system and cerebrovascular pulsatility). Another strength is that we systematically evaluated cognitive function using a comprehensive geriatric assessment, several neuropsychological tests, and blood biomarkers. Our findings may thus shed light on the mechanisms of cerebrovascular pulsatility, and encourage further exploration of the relationships between cerebral SVD and dementia.

The present study also has several limitations. A causal relationship between PWV and EPVS was unable to be established because of the study’s cross-sectional design. We were also unable to assess amyloid-β and α-synuclein in the current study because the Gerontological Investigation of Microbiome: a Longitudinal Estimation Study (the Gimlet study)^11^ did not perform cerebrospinal fluid testing or positron emission tomography. Furthermore, other studies have used different MMSE cut-off scores (e.g., MMSE < 26)^36^ to detect the presence of cognitive impairment. We also did not assess gray matter volume in the brain, which can be used to identify DLB subtypes^37^. Additionally, the combined inclusion of participants with DLB and those without dementia in our analyses of PWV and EPVS may have led to an overestimation of our interpretations. Moreover, the small number of participants with DLB and the large number of potential variables may have led to our study being statistically underpowered. Specifically, the small number of events per variable may have influenced the validity of the logistic model^38^. Selection bias may also exist because this was a single hospital-based cohort study in which participants agreeing to a fecal examination—the results of which were not used in the current study—were enrolled.

In conclusion, although this sub-study was a preliminary analysis, it provides evidence for relationships between EPVS, PWV, and DLB. BG-PVS was independently associated with DLB, and high PWV was associated with both BG-PVS and CS-PVS. High PWV may cause cerebrovascular pulsatility, which then accelerates EPVS and worsens cognitive impairment. Detailed assessments of the relationships between EPVS and PWV in DLB should be conducted in future studies to determine the underlying mechanisms.

## Methods

### Study design

We cross-sectionally performed a sub-analysis of data from a hospital-based prospective cohort study, the Gimlet study^10–12^, conducted at the National Center for Geriatrics and Gerontology (NCGG) in Japan. Briefly, patients who visited the Memory Clinic at the NCGG and agreed to undergo both a medical assessment of their cognitive function and a fecal examination were enrolled in the Gimlet study. This study was conducted in accordance with the principles of the Declaration of Helsinki and was approved by the Institutional Review Board of the NCGG (no. 1669). Written informed consent was obtained from all patients and their families before their participation in the Gimlet study. The Gimlet study is registered on the UMIN Clinical Trials Registry (UMIN000031851). Detailed information is provided in the Supplementary Materials.

### Participants

Between 2016 and 2017, we enrolled 128 participants who had subjective memory impairment to the Gimlet study, as previously reported^11^. In 2022, 22 participants presenting with either DLB, MCI, or NC were additionally enrolled to the Gimlet study to assess the relationship between DLB and gut microbiota. Participants in the Gimlet study were eligible for this MRI sub-study if they met the following criteria: (1) underwent brain MRI; and (2) were categorized as having either DLB, MCI, or NC. Of the 150 participants in the Gimlet study, 40 participants with dementia other than DLB and one participant with incomplete data were excluded. Thus, 109 participants were registered to this sub-analysis.

### Assessments

All participants underwent a comprehensive geriatric assessment based on the following features, as previously reported^11^: (1) demographic characteristics; (2) risk factors of cognitive impairment, such as hypertension and diabetes mellitus; (3) activities of daily living; (4) global cognitive function, assessed using the MMSE and CDR; (5) neuropsychological tests; (6) behavioral and psychological symptoms; (7) depression status; (8) laboratory parameters, such as NfL; (9) the ankle-brachial index as an indicator of arterial stiffness, and PWV as an indicator of arteriosclerosis^23^ and the impact of pulse^25^; and (9) nutritional and diet assessments^14^. Clinical samples and data were provided by the NCGG Biobank, which collects clinical data for research.

### Brain imaging

Patients underwent 1.5 T MRI (Philips Ingenia, Eindhoven, Netherlands) of the brain. The obtained MRI scans included diffusion-weighted imaging, fluid-attenuated inversion recovery (FLAIR) imaging, T2-weighted imaging, T2^*^-weighted gradient-echo imaging, three-dimensional T1-weighted sagittal and axial coronal views, and three-dimensional time-of-flight magnetic resonance angiography scans. VSRAD software (Eisai Co., Ltd., Tokyo, Japan) was used to quantify cortical and hippocampal atrophy; medial temporal structures involving the entire region of the entorhinal cortex, hippocampus, and amygdala show significant atrophy in patients with very mild AD, and can be specifically identified by a VSRAD software program^39^. Lower VSRAD values indicate less brain atrophy. One trained neurologist (N.S.) assessed all MR images and was blinded to participant data.

### Cerebral SVD

The presence and components of cerebral SVD were categorized according to the Standards for Reporting Vascular Changes on Neuroimaging recommendations^4^. We defined an SLI as a focal lesion of ≥ 3 mm in diameter that was hyperintense on T2-weighted imaging and hypointense on FLAIR images. We defined WMH as an irregular periventricular hyperintensity (Fazekas grade ≥ 3) and/or early confluent or confluent separate deep hyperintense lesions (Fazekas grade ≥ 2) in the white matter on T2-weighted and FLAIR images. We defined a CMB as a focal area of signal loss in the brain parenchyma of < 5 mm on a T2*-weighted gradient-echo imaging scan. We defined EPVS as small (< 3 mm), punctate (if perpendicular to the plane of the scan) or linear (if longitudinal to the plane of the scan) hyperintensities on T2 images in the basal ganglia or centrum semiovale, as previously reported^34^. BG-PVS and CS-PVS were coded using the following scale, which was applied to standard axial images: 0 (no EPVS); 1 (< 10 EPVS); 2 (11–20 EPVS); 3 (21–40 EPVS); and 4 (> 40 EPVS)^40^.

### Classification of cognitive function

Dementia was defined as an MMSE score < 20 and/or a CDR score ≥ 1, in accordance with the definitions used in our previous studies^10–12^. Participants who did not have dementia were further categorized as having either MCI or NC. MCI was defined as an MMSE score ≥ 20 and a CDR score of 0.5, which indicates possible, very mild dementia and a higher risk of developing dementia^10^. NC was defined as an MMSE score ≥ 20 and a CDR score of 0. Participants presenting with either MCI or NC were categorized into the non-dementia group in this sub-analysis.

### Diagnosis with DLB

DLB was diagnosed according to criteria developed by the DLB Consortium^8^. To assess DLB-related motor and non-motor symptoms, UPDRS^41^ scores and subscales were assessed by one trained neurologist (N.S.).

### Measurement of NfL

Blood samples were collected and frozen at − 81 °C in the NCGG Biobank. NfL was assessed as a disease-nonspecific biomarker of neural damage. Plasma NfL concentrations were measured using the NF-Light Advantage Kit on a highly sensitive single-molecule array assay Simoa HD-1 platform (Quanterix, Lexington, MA, USA) according to the manufacturer’s instructions^42^. All samples were measured blinded.

### Statistical analysis

Continuous, ordinal, and categorical variables are expressed as means ± standard deviations, medians and interquartile ranges, and frequencies and proportions (percentages), respectively. These data were compared using Student’s unpaired *t*-tests, Wilcoxon rank-sum tests, and χ^2^ tests, respectively. We first compared clinical characteristics between women and men, and between participants with DLB and those without dementia. Second, we compared clinical characteristics between participants with EPVS and those without EPVS. In detail, we prespecified a dichotomized classification of BG-PVS as high (score ≥ 2) or low (score < 2) and of CS-PVS as high (score ≥ 3) or low (score < 3) according to the distributions of the participants in the present study and the categories used in previous studies^43,44^. Third, the relationships between BG-PVS, CS-PVS, and PWV were evaluated separately. Finally, univariate and multivariate logistic regression models were used to identify independent associations between: (1) DLB and EPVS (both BG-PVS and CS-PVS), and (2) EPVS and PWV. The ORs and 95% CIs were calculated. All comparisons were two-tailed, and *p* < 0.05 represented statistical significance. Data were analyzed using the JMP 18.0 software package (SAS Institute Inc., Cary, NC, USA).

### Supplementary Information

Supplementary Information.

## Data Availability

The datasets used and/or analyzed during the current study are available from the corresponding author on reasonable request.
