# Supplementary material for: High pulse wave velocity is associated with enlarged perivascular spaces in dementia with Lewy bodies
Source: Sci Rep. 2024 Jun 17;14:13911. doi: 10.1038/s41598-024-64984-8 (PMC11183205; doi:10.1038/s41598-024-64984-8)
Supplement: Supplementary file 1 — Supplementary Information. [file 41598_2024_64984_MOESM1_ESM.docx]

**Supplementary file**

**High pulse wave velocity is associated with**

**enlarged perivascular spaces in dementia with Lewy bodies**

Naoki Saji, MD, PhD^a^, Yoshino Kinjo, MD, PhD^a,b^, Kenta Murotani, PhD^c^,

Shumpei Niida, PhD^d^, Akinori Takeda, MD, PhD^a^, Takashi Sakurai, MD, PhD^a,e,f^

1. Center for Comprehensive Care and Research on Memory Disorders, Hospital, National Center for Geriatrics and Gerontology, Aichi, Japan
2. Department of Cardiovascular Medicine, Nephrology and Neurology, University of the Ryukyus Graduate School of Medicine, Nishihara-cho, Okinawa, Japan.
3. Biostatistics Center, Graduate School of Medicine, Kurume University, Fukuoka, Japan
4. Core Facility Administration, Research Institute, National Center for Geriatrics and Gerontology, Aichi, Japan
5. Department of Prevention and Care Science, Research Institute, National Center for Geriatrics and Gerontology, Aichi, Japan
6. Department of Cognition and Behavioral Science, Nagoya University Graduate School of Medicine, Aichi, Japan

**Correspondence to:** Naoki Saji, M.D., Ph.D., Center for Comprehensive Care and Research on Memory Disorders, National Center for Geriatrics and Gerontology, 7-430, Morioka, Obu, Aichi 474-8511, Japan.

Tel.: +81 562 46 2311; Fax: +81 562 48 2373; E-mail: sajink@nifty.com

**Supplementary methods**

***Outline of the Gimlet study***

***Subjects***

Between March 2016 and March 2017, we enrolled 128 patients visiting the memory clinic at the National Center for Geriatrics and Gerontology (NCGG) in Japan who agreed to undergo a medical assessment of their cognitive function and a faecal examination. Patients were eligible for the Gimlet study if they met the following criteria: (1) were able to undergo a brain MRI; (2) provided informed consent in writing; (3) provided informed consent for the NCGG Biobank to store their clinical data, blood, and faecal samples; and (4) were accompanied by a study partner who could assess the patient’s condition. Patients were excluded if they met the following criteria: (1) were unable to undergo an MRI examination or the MRI was unable to be evaluated because of movement; (2) had local lesions, such as cerebral infarctions, that were detected by MRI before enrolment and might significantly affect cognitive function; (3) had a history of a major psychological disorder or current, serious, or unstable alcohol or drug abuse; (4) had ≤6 years of education; (5) had a history of cancer of the digestive tract; or (6) were judged by an investigator to be ineligible to participate as a study subject (e.g., recent use of antibiotics, brain tumour, encephalitis/meningitis, normal pressure hydrocephalus, or Huntington’s disease). In 2022, 22 participants presenting with either DLB, mild cognitive impairment (MCI), or normal cognition (NC) were additionally enrolled to the Gimlet study to assess the relationship between DLB and gut microbiota.

***Baseline assessment***

All participants underwent a comprehensive geriatric assessment^1^ using the following: (1) demographic characteristics; (2) risk factors, such as hypertension, dyslipidaemia, diabetes mellitus, ischaemic heart disease, chronic kidney disease, smoking habits, or a history of stroke and alcohol consumption; (3) basic and instrumental activities of daily living (ADL) scales, assessed using the Barthel Index^2^  and Lawton and Brody scale^3^; (4) global cognitive function, assessed using the Mini-Mental State Examination (MMSE)^4^ and Clinical Dementia Rating (CDR)^5^; (5) neuropsychological testing, using the ADAS-cog^6^, RCPM^7^, FAB^8^, and LM-WMSR^9^; (6) behavioural and psychological symptoms, assessed using the Dementia Behaviour Disturbance Scale (DBDS)^10^; (7) burden of caregivers, assessed by the Zarit Caregiver Burden Interview (ZBI)^11^; (8) depressive status, assessed by the Geriatric Depression Scale (GDS)^12^; (9) laboratory variables, including APOE ε4 as a risk factor for AD; (10) ankle brachial index (ABI) and pulse wave velocity (PWV), as indicators of arteriosclerosis^13^ and the ‘impact’ of pulse^14^; (11) brain imaging, such as MRI and SPECT; (12) an assessment of other factors, such as the presence of frailty^15^ and subjective hearing loss; and (13) an assessment of social and lifestyle factors, such as the Mini-Nutritional Assessment-Short Form (MNA-SF) to assess nutritional status^16^. All clinical samples and data were provided by the NCGG Biobank, which collects clinical data for research.

***Risk factors***

Hypertension was defined by a systolic blood pressure of ≥140 mmHg or a diastolic blood pressure of ≥90 mmHg, and/or the use of anti-hypertensive drugs. Dyslipidaemia was defined by a serum low-density lipoprotein cholesterol concentration of ≥140 mg/dL, a serum high-density lipoprotein cholesterol concentration of <40 mg/dL, a serum triacylglycerol concentration of ≥150 mg/dL, and/or the use of statins. Diabetes mellitus was defined by a haemoglobin A1c concentration of ≥6.5%, and/or the use of oral hypoglycaemic drugs or insulin, and/or a fasting serum glucose concentration of ≥69.9 mol/L (126 mg/dL). Ischaemic heart disease was defined by a history of physician-diagnosed angina pectoris and/or evidence of a prior myocardial infarction or coronary revascularisation procedure (percutaneous coronary intervention or coronary artery bypass surgery). Serum creatinine was measured and the estimated glomerular filtration rate (eGFR) was determined using the equation proposed by the Japanese Society of Nephrology, as follows: eGFR (mL/min/1.73 m^2^) = 194 × (serum creatinine [mg/dL])^−1.094^ × (age [years])^−0.287^ (× 0.739 if female). Chronic kidney disease (CKD) was defined by an eGFR of <60 mL/min/1.73 m^2^.

***MRI imaging***

Patients underwent a 1.5 T MRI (Philips Ingenia, Eindhoven, Netherlands) of the brain. In case of 1.5 T scanner was unavailable, 3.0 T MRI (Siemens AG Skyra, Muenchen, Germany) was performed. MRI scans were obtained, including diffusion-weighted images, fluid-attenuated inversion recovery images, T2-weighted images, T2^*^-weighted gradient-echo images, three-dimensional T1-weighted sagittal and axial coronal views, and 3D time-of-flight magnetic resonance angiography scans. The presence and components of cerebral small vessel disease, such as silent lacunar infarct (SLI), white matter hyperintensity (WMH), cerebral microbleeds (CMB), and enlarged periventricular space (EPVS), were categorized using previously published standards for reporting vascular changes on neuroimaging^17^. The voxel-based specific regional analysis system for Alzheimer’s Disease (VSRAD) software (Eisai Co., Ltd., Tokyo, Japan) was used to quantify cortical and hippocampal atrophy, using standardized z-scores^18^. A high VSRAD score suggests the presence of Alzheimer's disease (AD) because this score reflects hippocampal atrophy, which is one of the characteristics of the brain of a patient with AD. The participants also underwent N-isopropyl-p-[^123^I]-iodoamphetamine-SPECT, in which the presence of low blood flow in the area of the posterior cingulate gyrus and/or the precuneus was regarded as a surrogate marker of AD^19^.

***Sample collection***

Patients or their family members used a faecal sampler to collect a faecal sample as soon as possible after the patient’s bowel movement. The sample was placed in a specimen container. Patients collected faecal samples on the day of their hospital consultation, and the samples were presented to the clinical laboratory centre of the NCGG (preferably within 4 h of the bowel movement). Because one of the inclusion criteria for our study was that patients presented with study partners (family members), all of the demented patients were able to be supported if they needed support about excretion. Furthermore, when we received the faecal samples, we only accepted appropriate samples as per the analysing company’s criteria, while inadequate samples were disposed of and resubmission was requested. The samples were frozen and stored at −81°C in the NCGG Biobank. After all samples had been collected, they were transported (frozen) to TechnoSuruga Laboratory (Shizuoka, Japan).

***Gut microbiome***

Faecal samples were collected at home, just after a bowel evacuation, by patients or their family members. The samples were collected using scoop collection tubes, and patients consumed their usual diets before and after sample collection. Samples were frozen and preserved at –81°C at the NCGG Biobank. After all samples had been collected, the gut microbiomes were analysed by TechnoSuruga Laboratory (Shizuoka, Japan) using T-RFLP analysis^20^. T-RFLP analysis is one of the most well-established and reliable 16S ribosomal RNA-based methods, especially when considering its high throughput and reproducibility. First, T-RFLP was used to classify gut microbes into the following 10 groups: *Prevotella*, *Bacteroides*, Lactobacillales, *Bifidobacterium*, *Clostridium* cluster IV, *Clostridium* subcluster XIVa, *Clostridium* cluster IX, *Clostridium* cluster XI, *Clostridium* cluster XVIII, and ‘others’. Second, by referencing the Human Faecal Microbiome T-RFLP profile^21,22^, the gut microbiome was stratified into three enterotypes: enterotype I included *Bacteroides* at > 30%, enterotype II included *Prevotella* at > 15%, and enterotype III included the remaining bacteria. Third, the F/B ratio was examined, because an increase in F/B ratio indicates dysbiosis^23^. The phylum Firmicutes included the Lactobacillales and *Clostridium* clusters, and the phylum Bacteroidetes included *Bacteroides* and *Prevotella*.

***Analysis of metabolites in faeces***

To determine metabolite levels, we measured faecal levels of organic acids, SCFAs, ammonium ions, indoles, phenol, skatole, and p-cresol. The organic acids and SCFAs, such as acetic acid, propionic acid, butyric acid, iso-butyric acid, succinic acid, lactic acid, formic acid, valeric acid, and iso-valeric acid, were measured using high-performance liquid chromatography (Prominence, Shimadzu, Kyoto, Japan) with a detector (CDD-10A, Shimadzu, Kyoto, Japan), two tandemly-arranged columns (Shim-pack SCR-102(H), 300 mm × 8 mm ID, Shimadzu), and a guard column (Shim-pack SCR-102(H), 50 mm × 6 mm ID, Shimadzu). We measured ammonium ion concentrations using an ion chromatography system (ICS-1000, DIONEX, Thermo Fisher Scientific K.K., Tokyo, Japan) with a column (IonPac CS12A, 4 mm × 250 mm, DIONEX) and a guard column (IonPac CG12A, 4 mm × 50 mm, DIONEX). Indoles, phenol, skatole, and p-cresol levels were measured using gas chromatography/mass spectrometry (QP-2010, Shimadzu) and a capillary column (Inert cap WAX, 30 m × 0.25 mm × 0.25 µm, GL Sciences, Tokyo, Japan).

*Organic acids*

To determine the presence of organic acids, 0.1 g of faeces was placed into a 2.0-mL tube with zirconia beads and suspended in MilliQ water. Samples were heated at 85°C for 15 min, vortexed at 5 m/s for 45 s using FastPrep 24 (MP Biomedicals, CA, USA), and centrifuged at 15,350 × *g* for 10 min. The supernatant was filtered using a 0.2-µm filter. Organic acids (acetic acid, propionic acid, butyric acid, iso-butyric acid, succinic acid, lactic acid, formic acid, valeric acid, and iso-valeric acid) in faeces were measured using high-performance liquid chromatography (Prominence, Shimadzu) with a detector (CDD-10A, Shimadzu), two tandemly-arranged columns (Shim-pack SCR-102(H), 300 mm × 8 mm ID, Shimadzu), and a guard column (Shim-pack SCR-102(H), 50 mm × 6 mm ID, Shimadzu). A 5 mM p-toluenesulfonic acid solution was used in the mobile phase and a mixture (5 mM p-toluenesulfonic acid, 100 µM EDTA, and 20 mM Bis-Tris) was used as the reaction solution. The flow rate and oven temperature were 0.8 mL/min and 45°C, respectively. The detector cell temperature was maintained at 48°C. The measurements were performed with an absolute calibration curve (range 5–1,000 mg/L).

*Ammonia*

To measure ammonium ion concentrations, 0.1 g of faeces was suspended in 0.9 mL MilliQ water, heated at 85°C for 15 min, and filtered through a 0.20-µm filter. Samples were diluted 200-fold in MilliQ water. Ammonium ion concentrations were then measured using an ion chromatography system (ICS-1000, DIONEX) with a column (IonPac CS12A, 4 mm × 250 mm, DIONEX) and a guard column (IonPac CG12A, 4 mm × 50 mm, DIONEX). In the mobile phase, a 10.7 mM sulfuric acid solution was used, and the flow rate was 1.0 mL/min. The detector temperature was maintained at 35°C. The measurements were performed with an absolute calibration curve (range 0.025–2.0 mg/L).

*Indole, phenol, skatole, and p-cresol*

A total of 0.1 g of faeces was suspended in 2.5 mL phosphate buffer, including 0.4 mg/L 4-isopropylphenol as an internal standard. The sample was heated at 85°C for 15 min, mixed with 2.5 mL acetonitrile and 1 g NaCl, shaken for 30 min, and centrifuged at 1,300 × *g* for 10 min. Next, 1 mL of the supernatant was dehydrated and purified using a sodium sulphate drying cartridge (Bond Elut LRC, Agilent Technologies, USA), C18 cartridge (Smart SPE C18-30, AiSTI SCIENCE, Japan), and PSA cartridge (Smart SPE PSA-30, AiSTI SCIENCE), and placed into a vial. Indoles and phenols were measured using gas chromatography/mass spectrometry (QP-2010, Shimadzu) and a capillary column (Inert cap WAX, 30 m × 0.25 mm × 0.25 µm, GL Sciences). Helium was used as the carrier gas at 1.11 mL/min. The injector and interface temperatures were maintained at 240°C and 230°C, respectively. The oven temperature program was as follows: 70°C for 2 min; rose by 20°C/min to 200°C, held for 3 min; rose by 10°C/min to 240°C; and finally held for 16 min at 240°C. One microliter of the extract was injected in the splitless mode. The mass spectrometer was operated in electron impact ionisation mode at 70 eV. The measurements were performed with an absolute calibration curve (range 0.02–5.0 mg/L). Data acquisition was performed in selected ion monitoring mode for quantification.

**Supplementary tables.**

| **Table S1. Comparisons of background information between participants with DLB and those without dementia** | | | |
| --- | --- | --- | --- |
|  | DLB (+) | DLB (-) | *P* |
|  | (*n* = 16) | (*n* = 93) |  |
| ***Demographics*** |  |  |  |
| Age, years | 79, 73−82 | 76，68−80 | 0.092 |
| Sex, female, *n* (%) | 5 (31.3) | 48 (51.6) | 0.178 |
| Education, years | 12, 9−16 | 12，9−13 | 0.997 |
| Body mass index, kg/m^2^ | 22.5, 19.3−24.5 | 22.9, 21.2−25.2 | 0.321 |
| ***Risk factors*** |  |  |  |
| Hypertension, *n* (%) | 14 (87.5) | 53 (57.0) | 0.025 |
| Diabetes mellitus, *n* (%) | 5 (31.3) | 12 (12.9) | 0.127 |
| Dyslipidaemia, *n* (%) | 11 (68.8) | 43 (46.2) | 0.112 |
| IHD, *n* (%) | 0 | 10 (10.8) | 0.352 |
| Stroke, *n* (%) | 3 (18.8) | 5 (5.4) | 0.092 |
| CKD, *n* (%) | 4 (25.0) | 24 (25.8) | 1.000 |
| Smoking habits, *n* (%) | 2 (12.5) | 24 (25.8) | 0.349 |
| Alcohol consumption, *n* (%) | 5 (31.3) | 38 (40.9) | 0.585 |
| APOE ε4 carrier, *n* (%) | 6 (37.5) | 21 (22.6) | 0.219 |
| ***Comprehensive geriatric assessment*** | | | |
| Barthel Index | 88, 68−100 | 100, 100−100 | <.0001 |
| IADL impairment, *n* (%) | 13 (81.3) | 28 (30.1) | <.001 |
| DBDS | 12, 9–23 | 6, 3–11 | 0.001 |
| GDS | 4, 2–6 | 2, 1–4 | 0.213 |
| Vitality index | 9, 8–10 | 10, 9–10 | 0.072 |
| History of fall in a year | 11 (73.3) | 31 (34.4) | 0.009 |
| Gait speed, m/s | 1.06, 0.93−1.14 | 1.12, 0.89−1.29 | 0.357 |
| MNA-SF | 9, 6−9 | 13, 11−13 | <.0001 |
| ***Cognitive function*** |  |  |  |
| MMSE | 22, 17−24 | 27, 23−29 | <.0001 |
| CDR-SB | 4, 2−10 | 1, 0.5−2 | <.0001 |
| ADAS-cog | 13, 9.8–18.1 | 7, 4.7–10.4 | 0.001 |
| RCPM | 23, 19–29 | 29, 26–32 | 0.013 |
| FAB | 10, 4.8–11.3 | 12, 10–14 | 0.006 |
| LM-WMSR I | 5, 0–8 | 12, 7–19 | 0.002 |
| LM-WMSR II | 1, 0–3 | 5, 1–12 | 0.007 |
| ***Brain MRI findings*** |  |  |  |
| SLI, *n* (%) | 3 (18.8) | 4 (4.3) | 0.064 |
| WMH, *n* (%) | 4 (25.0) | 23 (24.7) | 1.000 |
| CMB, *n* (%) | 5 (31.3) | 14 (15.1) | 0.150 |
| BG-PVS ≧2, *n* (%) | 10 (62.5) | 23 (24.7) | 0.006 |
| CS-PVS ≧3, *n* (%) | 5 (31.3) | 33 (35.5) | 1.000 |
| VSRAD | 1.05, 0.90–1.57 | 0.84, 0.56–1.26 | 0.020 |
| ***Arterial stiffness*** |  |  |  |
| Ankle brachial index | 1.07, 1.00−1.17 | 1.12, 1.07−1.15 | 0.168 |
| Pulse wave velocity, m/s | 19.9, 17.7−21.4 | 17.9, 15.7−22.0 | 0.189 |
| ***Laboratory findings*** |  |  |  |
| BNP, pg/mL | 40, 31.1−85.0 | 29.0，14.4−59.5 | 0.042 |
| NfL, pg/mL | 31.4, 21.7−59.0 | 21.3, 15.0−26.3 | 0.001 |

Data are presented as medians, interquartile ranges or number of patients (%). The Wilcoxon rank-sum test and χ^2^ test were used.

Note that participants without dementia presented with normal cognition or mild cognitive impairment.

Abbreviations: ADAS-cog, Alzheimer’s Disease Assessment Scale-Cognitive Subscale; APP, Amyloid Precursor Protein; APOE, apolipoprotein E; BG-PVS, enlarged perivascular spaces in the basal ganglia; BNP, Brain Natriuretic Peptide; BP, blood pressure; CDR-SB, Clinical Dementia Rating-Sum of Boxes; CMB, cerebral microbleed; CKD, chronic kidney disease; CS-PVS, enlarged perivascular spaces in the centrum semiovale; DBDS, Dementia Behavior Disturbance Scale; DLB, dementia with Lewy bodies; EPVS, enlarged periventricular space; FAB, Frontal Assessment Battery; GDS, Geriatric Depression Scale; IADL, instrumental activities of daily living; IHD, ischemic heart disease; JDI, Japanese diet index; LM-WMSR, Logical Memory subtests I and II of the Wechsler Memory Scale-Revised; MMSE, Mini-Mental State Examination; MNA-SF, Mini-Nutritional Assessment-Short Form; MRI, magnetic resonance imaging; NfL, Neurofilament light chain; RCPM, Raven’s Coloured Progressive Matrices; SLI, silent lacunar infarct; VSRAD, voxel-based specific regional analysis system for Alzheimer’s disease; WMH, white matter hyperintensity.

| **Table S2. Comparisons of background information between participants with high and low BG-PVS scores** | | | |
| --- | --- | --- | --- |
|  | BG-PVS ≥ 2 | BG-PVS < 2 | *P* |
|  | (*n* = 33) | (*n* = 76) |  |
| ***Demographics*** |  |  |  |
| Age, years | 79, 76−82 | 75, 67−79 | < 0.001 |
| Sex, female, *n* (%) | 14 (42.4%) | 39 (51.3%) | 0.413 |
| Education, years | 12, 9−12 | 12，9−14 | 0.136 |
| Body mass index, kg/m^2^ | 23.3, 21.3−26.0 | 22.6, 20.7−24.6 | 0.321 |
| Systolic BP, mmHg | 150, 131−164 | 142, 124−162 | 0.200 |
| Diastolic BP, mmHg | 81, 73−92 | 81, 70−89 | 0.812 |
| ***Risk factors*** |  |  |  |
| Hypertension, *n* (%) | 25 (75.8) | 42 (55.3) | 0.055 |
| Diabetes mellitus, *n* (%) | 8 (24.2) | 9 (11.8) | 0.149 |
| Dyslipidaemia, *n* (%) | 19 (57.6) | 35 (46.1) | 0.302 |
| IHD, *n* (%) | 4 (12.1) | 6 (7.9) | 0.487 |
| Stroke, *n* (%) | 6 (18.2) | 2 (2.6) | 0.009 |
| CKD, *n* (%) | 6 (18.2) | 22 (29.0) | 0.340 |
| Smoking habits, *n* (%) | 4 (12.1) | 22 (29.0) | 0.086 |
| Alcohol consumption, *n* (%) | 10 (30.3) | 33 (43.4) | 0.286 |
| APOE ε4 carrier, *n* (%) | 10 (30.3) | 17 (22.4) | 0.470 |
| ***Comprehensive geriatric assessment*** | | | |
| Barthel Index | 100, 86−100 | 100, 100−100 | 0.005 |
| IADL impairment, *n* (%) | 20 (60.6) | 21 (27.6) | 0.002 |
| DBDS | 11, 5−19 | 6, 3−10 | 0.001 |
| GDS | 3, 0−5 | 3, 1−5 | 0.503 |
| Vitality index | 9, 9−10 | 10, 9−10 | 0.032 |
| History of fall in a year, *n* (%) | 16 (50.0) | 26 (35.6) | 0.197 |
| Gait speed, m/s | 1.01, 0.70−1.19 | 1.13, 0.96−1.30 | 0.066 |
| MNA-SF | 10, 9−13 | 12, 10−13 | 0.080 |
| ***Cognitive function*** |  |  |  |
| MMSE | 24, 22−27 | 27, 23−29 | 0.031 |
| CDR-SB | 2, 1−4 | 1, 0.5−2 | 0.001 |
| ADAS-cog | 9.3, 5−13 | 7, 4.7−10.4 | 0.130 |
| RCPM | 26, 22−30 | 30, 27−33 | 0.002 |
| FAB | 10, 9−12 | 13, 10−14 | <0.001 |
| LM-WMSR I | 9, 4.5−14 | 12, 7−20 | 0.072 |
| LM-WMSR II | 3, 1−7 | 5, 1−13 | 0.152 |
| ***Brain MRI findings*** |  |  |  |
| SLI, *n* (%) | 5 (15.2) | 2 (2.6) | 0.026 |
| WMH, *n* (%) | 9 (27.3) | 18 (23.7) | 0.810 |
| CMB, *n* (%) | 8 (24.2) | 11 (14.5) | 0.273 |
| CS-PVS ≧3, *n* (%) | 19 (57.6) | 19 (25.0) | 0.002 |
| VSRAD | 1.01, 0.77−1.21 | 0.84, 0.55−1.60 | 0.197 |
| ***Arterial stiffness*** |  |  |  |
| Ankle brachial index | 1.08, 1.05−1.15 | 1.13, 1.07−1.16 | 0.204 |
| Pulse wave velocity, m/s | 20.3, 18.2−23.4 | 17.3, 15.1−21.1 | 0.010 |
| ***Laboratory findings*** |  |  |  |
| HbA1c, % | 5.9, 5.7−6.4 | 5.7, 5.5−6.0 | 0.042 |
| BNP, pg/mL | 34.5, 25.8−86.3 | 28.9, 14.0−49.9 | 0.013 |
| NfL, pg/mL | 26.1, 21.3−35.5 | 19.8, 14.4−25.0 | <0.001 |

Data are presented as medians, interquartile ranges, or number of patients (%). Wilcoxon rank-sum test and χ^2^ test were used.

Note that participants with high BG-PVS were defined as presenting with enlarged perivascular spaces in the basal ganglia (scores ≥ 2 based on an MRI scan at the level of the basal ganglia).

Abbreviations: ADAS-cog, Alzheimer’s Disease Assessment Scale-Cognitive Subscale; APP, Amyloid Precursor Protein; APOE, apolipoprotein E; BG-PVS, enlarged perivascular spaces in the basal ganglia; BNP, Brain Natriuretic Peptide; BP, blood pressure; CDR-SB, Clinical Dementia Rating-Sum of Boxes; CMB, cerebral microbleed; CKD, chronic kidney disease; CS-PVS, enlarged perivascular spaces in the centrum semiovale; DBDS, Dementia Behavior Disturbance Scale; DLB, dementia with Lewy bodies; EPVS, enlarged periventricular space; FAB, Frontal Assessment Battery; GDS, Geriatric Depression Scale; IADL, instrumental activities of daily living; IHD, ischemic heart disease; JDI, Japanese diet index; LM-WMSR, Logical Memory subtests I and II of the Wechsler Memory Scale-Revised; MMSE, Mini-Mental State Examination; MNA-SF, Mini-Nutritional Assessment-Short Form; MRI, magnetic resonance imaging; NfL, Neurofilament light chain; RCPM, Raven’s Coloured Progressive Matrices; SLI, silent lacunar infarct; VSRAD, voxel-based specific regional analysis system for Alzheimer’s disease; WMH, white matter hyperintensity.

| **Table S3. Comparisons of background information between participants with high and low CS-PVS scores** | | | |
| --- | --- | --- | --- |
|  | CS-PVS ≥ 3 | CS-PVS < 3 | *P* |
|  | (n = 38) | (n = 71) |  |
| ***Demographics*** |  |  |  |
| Age, years | 77, 74−80 | 75，68−81 | 0.146 |
| Sex, female, *n* (%) | 17 (44.7) | 36 (50.7) | 0.688 |
| Education, years | 12, 9−13 | 12, 9−13 | 0.658 |
| Body mass index, kg/m^2^ | 23.3, 21.3−26.1 | 22.6, 20.8−24.1 | 0.171 |
| ***Risk factors*** |  |  |  |
| Hypertension, *n* (%) | 22 (57.9) | 45 (63.4) | 0.680 |
| Diabetes mellitus, *n* (%) | 7 (18.4) | 10 (14.1) | 0.587 |
| Dyslipidaemia, *n* (%) | 15 (39.5) | 39 (54.9) | 0.160 |
| IHD, *n* (%) | 4 (10.5) | 6 (8.5) | 0.737 |
| Stroke, *n* (%) | 5 (13.2) | 3 (4.2) | 0.124 |
| CKD, *n* (%) | 11 (29.0) | 17 (23.9) | 0.647 |
| Smoking habits, *n* (%) | 4 (10.5) | 22 (31.0) | 0.019 |
| Alcohol consumption, *n* (%) | 13 (34.2) | 30 (42.3) | 0.538 |
| APoE ε4, *n* (%) | 11 (29.0) | 16 (22.5) | 0.491 |
| ***Comprehensive geriatric assessment*** | | | |
| Barthel Index | 100, 100−100 | 100, 100−100 | 0.290 |
| IADL impairment, *n* (%) | 19 (50.0) | 22 (31.0) | 0.063 |
| DBDS | 8, 5–14 | 6, 3–11 | 0.099 |
| GDS | 2, 1–4 | 3, 1–5 | 0.339 |
| Vitality index | 10, 9–10 | 10, 9–10 | 0.929 |
| History of fall in a year | 15 (40.5) | 27 (39.7) | 1.000 |
| Gait speed, m/s | 1.11, 0.72−1.27 | 1.12, 0.97−1.30 | 0.339 |
| MNA-SF | 13, 10−13 | 12, 10−13 | 0.492 |
| ***Cognitive function*** |  |  |  |
| MMSE | 26, 23−29 | 25, 23−29 | 0.868 |
| CDR-SB | 1.3, 0.5−2.6 | 1, 0.5−2.6 | 0.537 |
| ADAS-cog | 8.2, 4.2–11.4 | 7.6, 5.3–11.4 | 0.654 |
| RCPM | 29, 24–31.3 | 30, 25.5–33 | 0.215 |
| FAB | 11, 10–14 | 12, 10–14 | 0.178 |
| LM-WMSR I | 10, 6–18 | 11, 6–18 | 0.752 |
| LM-WMSR II | 3, 1–10.5 | 5, 1–11 | 0.551 |
| ***Brain MRI findings*** |  |  |  |
| SLI, *n* (%) | 2 (5.3) | 5 (7.0) | 1.000 |
| WMH, *n* (%) | 10 (26.3) | 17 (23.9) | 0.818 |
| CMB, *n* (%) | 7 (18.4) | 12 (16.9) | 1.000 |
| VSRAD | 0.94, 0.68–1.21 | 0.87, 0.57–1.63 | 0.953 |
| ***Arterial stiffness*** |  |  |  |
| Ankle brachial index | 1.08, 1.05−1.18 | 1.12, 1.07−1.15 | 0.594 |
| Pulse wave velocity, m/s | 20.4, 17.8−23.5 | 17.4, 15.6−20.5 | 0.006 |
| ***Laboratory findings*** |  |  |  |
| BNP, pg/mL | 30.2, 20.6−63.8 | 30，13.5−64.7 | 0.308 |
| NfL, pg/mL | 24.5, 20.9−31.0 | 20.7, 15.2−28.9 | 0.049 |

Data are presented as medians, interquartile ranges or number of patients (%). The Wilcoxon rank-sum test and χ^2^ test were used.

Note that participants with high CS-PVS were defined as presenting with enlarged perivascular spaces in the centrum semiovale (scores ≥ 3 based on an MRI scan at the level of the centrum semiovale).

Abbreviations: ADAS-cog, Alzheimer’s Disease Assessment Scale-Cognitive Subscale; APP, Amyloid Precursor Protein; APOE, apolipoprotein E; BG-PVS, enlarged perivascular spaces in the basal ganglia; BNP, Brain Natriuretic Peptide; BP, blood pressure; CDR-SB, Clinical Dementia Rating-Sum of Boxes; CMB, cerebral microbleed; CKD, chronic kidney disease; CS-PVS, enlarged perivascular spaces in the centrum semiovale; DBDS, Dementia Behavior Disturbance Scale; DLB, dementia with Lewy bodies; EPVS, enlarged periventricular space; FAB, Frontal Assessment Battery; GDS, Geriatric Depression Scale; IADL, instrumental activities of daily living; IHD, ischemic heart disease; JDI, Japanese diet index; LM-WMSR, Logical Memory subtests I and II of the Wechsler Memory Scale-Revised; MMSE, Mini-Mental State Examination; MNA-SF, Mini-Nutritional Assessment-Short Form; MRI, magnetic resonance imaging; NfL, Neurofilament light chain; RCPM, Raven’s Coloured Progressive Matrices; SLI, silent lacunar infarct; VSRAD, voxel-based specific regional analysis system for Alzheimer’s disease; WMH, white matter hyperintensity.

| **Table S4. Comparisons of background information between participants with high and low BG-PVS scores in participants with DLB** | | | |
| --- | --- | --- | --- |
|  | BG-PVS ≥ 2 | BG-PVS < 2 | *P* |
|  | (n = 10) | (n = 6) |  |
| ***Demographics*** |  |  |  |
| Age, years | 78, 75−82 | 79，70−83 | 1.000 |
| Sex, female, *n* (%) | 2 (20) | 3 (50) | 0.300 |
| ***MDS-UPDRS*** |  |  |  |
| UPDRS I, points | 18, 8−22 | 7, 6−11 | 0.106 |
| UPDRS II, points | 22, 7−32 | 7, 1−27 | 0.341 |
| UPDRS III, points | 36, 10−56 | 23, 5−62 | 0.661 |
| ***Risk factors*** |  |  |  |
| Hypertension, *n* (%) | 8 (80.0) | 6 (100.0) | 0.500 |
| Diabetes mellitus, *n* (%) | 2 (20.0) | 3 (50.0) | 0.300 |
| Dyslipidaemia, *n* (%) | 7 (70.0) | 4 (66.7) | 1.000 |
| IHD, *n* (%) | 0 | 0 | - |
| Stroke, *n* (%) | 3 (30.0) | 0 | 0.250 |
| CKD, *n* (%) | 2 (20.0) | 2 (33.3) | 0.604 |
| Smoking habits, *n* (%) | 1 (10.0) | 1 (16.7) | 1.000 |
| Alcohol consumption, *n* (%) | 3 (30.0) | 2 (33.3) | 1.000 |
| ***Comprehensive geriatric assessment*** | | | |
| Barthel Index | 85, 64−100 | 100, 65−100 | 0.364 |
| IADL impairment, *n* (%) | 8 (80.0) | 5 (83.3) | 1.000 |
| DBDS | 19, 9–26 | 9, 8–12 | 0.125 |
| GDS | 4, 0–8 | 4, 2–6 | 0.902 |
| Vitality index | 9, 8–10 | 10, 9–10 | 0.152 |
| History of fall | 7 (87.5) | 2 (40.0) | 0.217 |
| Gait speed, m/s | 1.06, 0.95−1.15 | 1.05, 0.86−1.13 | 0.513 |
| MNA-SF | 9, 5−9 | 9, 7−10 | 0.659 |
| ***Cognitive function*** |  |  |  |
| MMSE | 22, 16−24 | 22, 16−23 | 0.870 |
| CDR-SB | 5, 2−12 | 3, 2−8 | 0.385 |
| ADAS-cog | 13.4, 8.5–20.4 | 12, 10.3–17.1 | 0.915 |
| RCPM | 23, 20–27 | 26, 13–33 | 0.915 |
| FAB | 8, 4–11 | 11, 8–13 | 0.195 |
| LM-WMSR I | 6, 0–12 | 4, 1–7 | 0.588 |
| LM-WMSR II | 1, 0–4 | 2, 0–3 | 0.740 |
| ***Brain MRI findings*** |  |  |  |
| SLI, *n* (%) | 3 (30.0) | 0 | 0.250 |
| WMH, *n* (%) | 3 (30.0) | 1 (16.7) | 0.551 |
| CMB, *n* (%) | 3 (30.0) | 2 (33.3) | 1.000 |
| VSRAD | 1.05, 0.92–1.36 | 1.19, 0.89–2.01 | 0.745 |
| ***Arterial stiffness*** |  |  |  |
| Ankle brachial index | 1.07, 1.03−1.16 | 1.07, 0.98−1.17 | 0.664 |
| Pulse wave velocity, m/s | 20.1, 18.6−22.9 | 18.2, 17.5−21.9 | 0.515 |
| ***Laboratory findings*** |  |  |  |
| BNP, pg/mL | 79.2, 39.1−117.7 | 34.3，25.3−41.6 | 0.046 |
| NfL, pg/mL | 36.4, 26.1−75.8 | 22.2, 16.1−47.9 | 0.083 |

Data are presented as medians, interquartile ranges, or number of patients (%). Wilcoxon rank-sum test and χ^2^ test were used.

Note that participants with high BG-PVS were defined as presenting with enlarged perivascular spaces in the basal ganglia (scores ≥ 2 based on an MRI scan at the level of the basal ganglia).

Abbreviations: ADAS-cog, Alzheimer’s Disease Assessment Scale-Cognitive Subscale; APP, Amyloid Precursor Protein; APOE, apolipoprotein E; BG-PVS, enlarged perivascular spaces in the basal ganglia; BNP, Brain Natriuretic Peptide; BP, blood pressure; CDR-SB, Clinical Dementia Rating-Sum of Boxes; CMB, cerebral microbleed; CKD, chronic kidney disease; CS-PVS, enlarged perivascular spaces in the centrum semiovale; DBDS, Dementia Behavior Disturbance Scale; DLB, dementia with Lewy bodies; EPVS, enlarged periventricular space; FAB, Frontal Assessment Battery; GDS, Geriatric Depression Scale; IADL, instrumental activities of daily living; IHD, ischemic heart disease; JDI, Japanese diet index; LM-WMSR, Logical Memory subtests I and II of the Wechsler Memory Scale-Revised; MMSE, Mini-Mental State Examination; MNA-SF, Mini-Nutritional Assessment-Short Form; MRI, magnetic resonance imaging; NfL, Neurofilament light chain; RCPM, Raven’s Coloured Progressive Matrices; SLI, silent lacunar infarct; UPDRS, Unified Parkinson's Disease Rating Scale; VSRAD, voxel-based specific regional analysis system for Alzheimer’s disease; WMH, white matter hyperintensity.

**References**

1. Toba K. The guideline for comprehensive geriatric assessment. *Nihon Ronen Igakkai Zasshi.***42**,177–80 (2005).
2. Mahoney FI, Barthel DW, Callahan JP. Rehabilitation of the hemiplegic patient: a clinical evaluation. *South Med J*. **48**, 472–80 (1955).
3. Lawton MP, Brody EM. Assessment of older people: Self-maintaining and instrumental activities of daily living. *Gerontologist Autumn*. **9**, 179–86 (1969).
4. Folstein MF, Folstein SE, McHugh PR. "Mini-mental state". A practical method for grading the cognitive state of patients for the clinician. *J Psychiatr Res*. **12**, 189–98 (1975).
5. Morris JC. The Clinical Dementia Rating (CDR): current version and scoring rules. *Neurology*. **43**, 2412–4 (1993).
6. Rosen WG, Mohs RC, Davis KL. A new rating scale for Alzheimer's disease. *Am J Psychiatry*. **141**, 1356–64 (1984).
7. Raven J. Guide to using the coloured progressive matrices. London. H.K. Lewis (1965).
8. Dubois B, Slachevsky A, Litvan I, Pillon B. The FAB: a Frontal Assessment Battery at bedside. *Neurology*. **55**, 1621–6 (2000).
9. Wechsler D. Wechsler memory scale-revised. San Antonio, TX. Psychological Corporation (1981).
10. Baumgarten M, Becker R, Gauthier S. Validity and reliability of the dementia behavior disturbance scale. *J Am Geriatr Soc*. **38**, 221–6 (1990).
11. Zarit SH, Reever KE, Bach-Peterson J. Relatives of the impaired elderly: correlates of feelings of burden. *Gerontologist*. **20**, 649–55 (1980).
12. Yesavage JA, et al. Development and validation of a geriatric depression screening scale: a preliminary report. *J Psychiatr Res*. **17**, 37–49 (1982).
13. Saji N*,* et al. Comparison of arteriosclerotic indicators in patients with ischemic stroke: ankle-brachial index, brachial-ankle pulse wave velocity and cardio-ankle vascular index. *Hypertens Res*. **38**, 323–8 (2015).
14. Saji N, Toba K, Sakurai T. Cerebral small vessel disease and arterial stiffness: tsunami effect in the brain? *Pulse*. **23**, 182–9 (2016).
15. Fried LP, et al. Cardiovascular Health Study Collaborative Research Group. Frailty in older adults: evidence for a phenotype. *J Gerontol A Biol Sci Med Sci.* **56**, M146–56 (2001).
16. Vellas B*, et al. O*verview of the MNA–Its history and challenges. *J Nutr Health Aging.* **10**, 456–63; discussion 463-5 (2006).
17. Wardlaw JM, et al. Neuroimaging standards for research into small vessel disease and its contribution to ageing and neurodegeneration. *Lancet Neurol.* **12**, 822–38 (2013).
18. Matsuda H, et al. Automatic voxel-based morphometry of structural MRI by SPM8 plus diffeomorphic anatomic registration through exponentiated lie algebra improves the diagnosis of probable Alzheimer Disease. *AJNR Am J Neuroradiol.* **33**, 1109–14 (2012).
19. Ito K, et al. Prediction of outcomes in MCI with (123)I-IMP-CBF SPECT: a multicenter prospective cohort study. *Ann Nucl Med*. **27**, 898–906 (2013).
20. Osborn AM, Moore ER, Timmis KN. An evaluation of terminal-restriction fragment length polymorphism (T-RFLP) analysis for the study of microbial community structure and dynamics. *Environ Microbiol.* **2**, 39–50 (2000).
21. Arumugam M, et al. Enterotypes of the human gut microbiome. *Nature*. **12**, 473:174–80 (2011).
22. Emoto T, et al. Analysis of Gut Microbiota in Coronary Artery Disease Patients: a Possible Link between Gut Microbiota and Coronary Artery Disease. *J Atheroscler Thromb*. **23**, 908–21 (2016).
23. Spychala MS, et al. Age-related changes in the gut microbiota influence systemic inflammation and stroke outcome. *Ann Neurol.* **84**, 23–36 (2018).
